# Figure Caption Correction: Characteristics of Articles About Human Papillomavirus Vaccination in Japanese Newspapers: Time-Series Analysis Study

**DOI:** 10.2196/10878

**Published:** 2018-05-10

**Authors:** Nao Ueda, Ryoki Yokouchi, Taro Onoda, Atsushi Ogihara

**Affiliations:** ^1^ Graduate School of Human Sciences Waseda University Tokorozawa Japan; ^2^ Department of Health Sciences and Social Welfare School of Human Sciences Waseda University Tokorozawa Japan; ^3^ Department of Medicine School of Medicine Tokai University Isehara Japan; ^4^ Faculty of Human Sciences Waseda University Tokorozawwa Japan

The authors of the paper “Characteristics of Articles About Human Papillomavirus Vaccination in Japanese Newspapers: Time-Series Analysis Study” (JMIR Public Health Surveill 2017;3(4):e97) omitted information in the caption of  Figure 2. It should have read “Worldwide trends in cervical cancer vaccination. HPV: human papillomavirus. (Modified by the authors based on Wilson et al [6]).”

The corrected caption appeared in the online version of the paper on the JMIR website on March 16, 2018, together with the publication of a correction notice (J Med Internet Res 2018;20(3):e27). Because this was made after submission to PubMed or Pubmed Central and other full-text repositories, the corrected article was also re-submitted to those repositories.

However, the original correction notice published on March 16, 2018, erroneously appeared in a different journal (J Med Internet Res) than the original paper (JMIR Public Health and Surveillance), and was retracted on May 10, 2018, together with republication of this correction in the correct journal.

Please see the figure with the corrected caption here.

**Figure 2:**
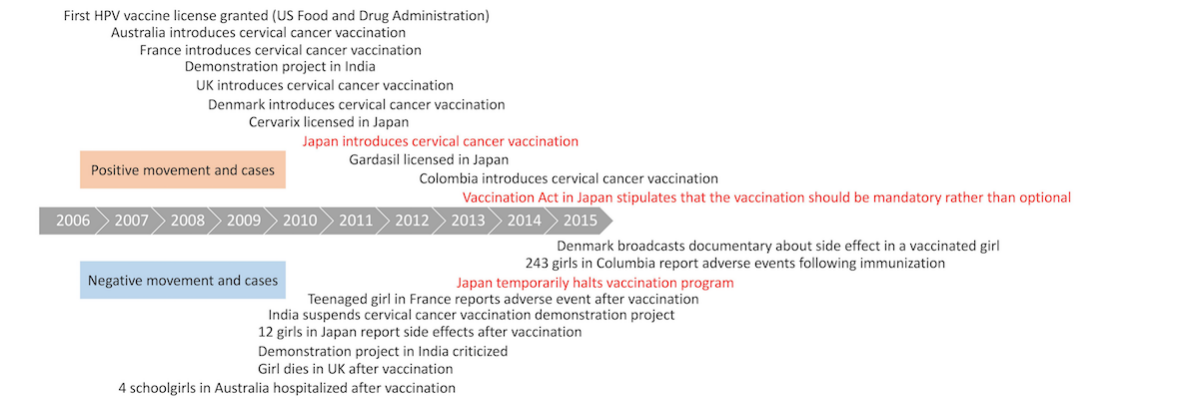
Worldwide trends in cervical cancer vaccination. HPV: human papillomavirus. (Modified by The authors based on Wilson et al).

